# Effect of crocin on cardiac antioxidants, and hemodynamic parameters after injuries induced by hepatic ischemia-reperfusion in rats

**DOI:** 10.22038/ijbms.2019.29660.7159

**Published:** 2019-03

**Authors:** Ghaidafeh Akbari, Seyyed Ali Mard, Mahin Dianat

**Affiliations:** 1 Department of Physiology, Yasuj University of Medical Sciences, Yasuj, Iran; 2 Alimentary Tract Research Center, Physiology Research Center, Department of Physiology, School of Medicine, Ahvaz Jundishapur University of Medical Sciences, Ahvaz, Iran; 3 Physiology Research Center, Department of Physiology, School of Medicine, Ahvaz Jundishapur University of Medical Sciences, Ahvaz, Iran

**Keywords:** Crocin, Heart, Hemodynamic parameters, Ischemia/reperfusion, Liver

## Abstract

**Objective(s):**

This research aimed to test the impact of liver ischemia/reperfusion (IR) insult on the activity of antioxidant enzymes, functional enzymes, histological, and hemodynamic parameters of heart, as well as protective function of crocin on these variables in rats.

**Materials and Methods:**

Thirty two rats were randomly assigned into 4 experimental groups (8 rats in each). I: sham-operated, II: IR induction, III: Crocin alone, and IV: Crocin+IR induction. Groups I and III received normal saline at 2 ml per day and crocin at 200 mg per kg on a daily basis for a week via intraperitoneally injection. Afterwards, laparotomy was performed. Groups II and IV was also received normal saline and crocin and then experienced a 45 min ischemia followed by 1 hr reperfusion. Tissue samples of heart and blood were taken to use for further microscopic and laboratories analysis. Hemodynamic parameters were measured by tail cuff method.

**Results:**

Findings indicated that crocin dramatically elevated the activity of antioxidant enzymes, and attenuated serum concentrations of hepatic and cardiac enzymes. Crocin also inhibited histopathological disarrangements, and modulated hemodynamic parameters beyond IR-induced hepatic insult.

**Conclusion:**

Current experiment indicated that crocin has potential cardioprotective action following hepatic I/R-induced damage. Therefore, it can be administered before elective hepatic surgeries.

## Introduction

Liver ischemia-reperfusion (IR) insult is an important side effect, which happens following liver graft, hapatectomy, and hepatic pedicle clamping during liver interventions (1). Myocardial damages have been shown to occur following major liver surgical procedures if the liver is subjected to a significant decrease in blood supply or ischemia followed by reperfusion (2) that may lead to oxidative stress through promoting the concentrations of malondialdehyde, and decreasing the activity of main antioxidant enzymes. It is well-known that antioxidants have beneficial impact against injuries induced by IR via reactive oxygen species (ROS) scavenging (3). 

Hemodynamic instability during reperfusion course has been attributed to hypervolemia, acute left ventricular failure, the release of myocardial depressants and concomitant decrease in left ventricular contractility that is characterized by hemodynamic changes such as decrease in mean arterial pressure and central venous pressure (2).

Recently, phytotherapy in developing community is considered as a remedy to manage health problems (4). Ingredients such as flavonoids and phenolic compounds in plants scavenge free radicals (5), by antioxidant activity (6).

**Figure 1:**
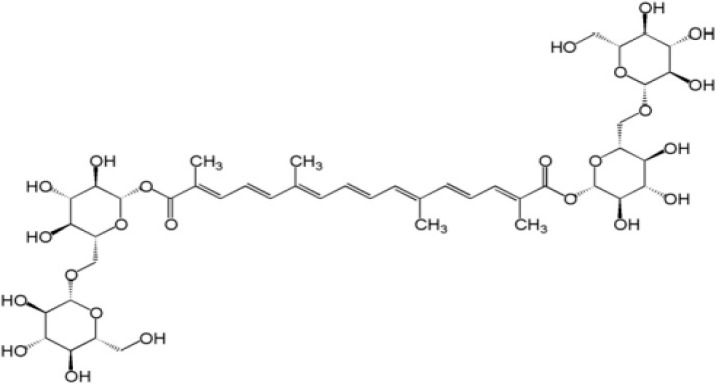
Chemical structure of crocin (C_44_H_64_O_24_)

Crocin (Figure 1) (with the chemical formula of C_44_H_64_O_24_) is one of the most important antioxidant compounds (7) of *Crocus sativus* Linn (saffron) (8). The hepato-(9), reno-(10), and retino-protective (11) actions of crocin on IR-induced damage have been reported. 

Thus, the benefits of crocin on cardiac IR-induced damage are well determined, while its effects against cardiac insult beyond hepatic IR-induced injury have not been specified. Therefore, the current experiment purposed to evaluate the effect of liver IR damage on the activity of antioxidant enzymes, functional enzymes, histological and hemodynamic parameters of heart, and protective function of crocin on these variables in rats. 

## Materials and Methods

***Animal***

 The animals used in this study were rat [Wistar, male] weighing 200–250 g. Rats purchased from animal center of Ahvaz Jundishapur University of Medical Sciences, Ahvaz, Iran. They were fed with a standard regimen and tap water ad libitum. All experiments including anesthesia, surgery, inducing IR, and killing were performed according to the regulations of ethics committee of Ahvaz Jundishapur University of Medical Sciences (RDC-9416). Animals were kept in an air-conditioned room with controlled temperature 22±2 ^°^C, and under 12/12 hr light/dark cycle. All rats were deprived of food overnight before the induction of I/R injury. 

***Experimental groups***

Thirty two rats were randomly grouped in 4 experimental groups (n=8); Sham-operated group: rats in this group were given physiologic saline 2 ml per day on a daily basis via intraperitoneally injection within one week before surgery (12). Then, they experienced a midline abdominal surgery without induction of I/R injury. IR group: this group of rats experienced the same protocol plus induction of IR injury. 

*Crocin pretreatment group*

Animals in this group received crocin at 200 mg per kg on a daily basis within a week via intraperitoneally route, and then experienced the same protocol performed for the sham-operated group (13).

*Crocin pretreatment+IR group*

Animals in this group received crocin at 200 mg per kg on a daily basis within a week via intraperitoneally route, and then experienced the same protocol performed for the IR group.

**Figure 2:**
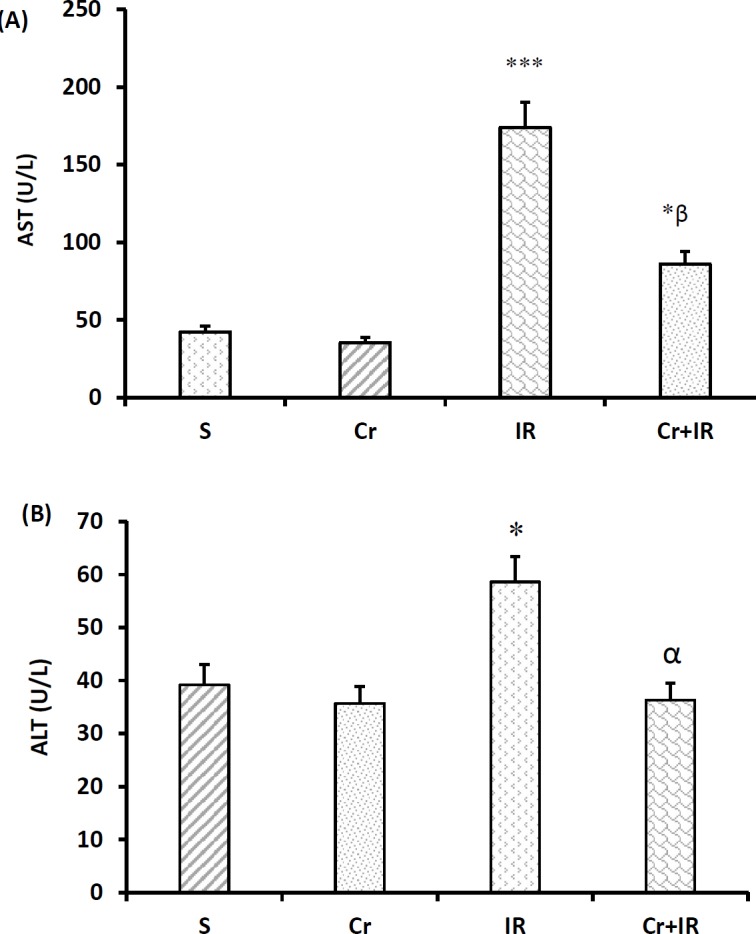
Crocin pretreatment at 200 mg/kg for a week once per day decreased serum levels of AST, and ALT beyond IR-induced liver injury. Data represented as mean±SEM, 8 rats in each group. *P<0.05 and ***P<0.001 significant difference compared to the sham-operated rats and α*P*<0.05 and β*P*<0.01 significant difference compared to the IR group. Cr: Crocin pretreatment; IR: Ischemia/reperfusion; S: Sham-operated rats; Cr +IR: Crocin pretreatment+ischemia/reperfusion; AST: Aspartate aminotransferase and ALT: Alanine aminotransferase

**Figure 3:**
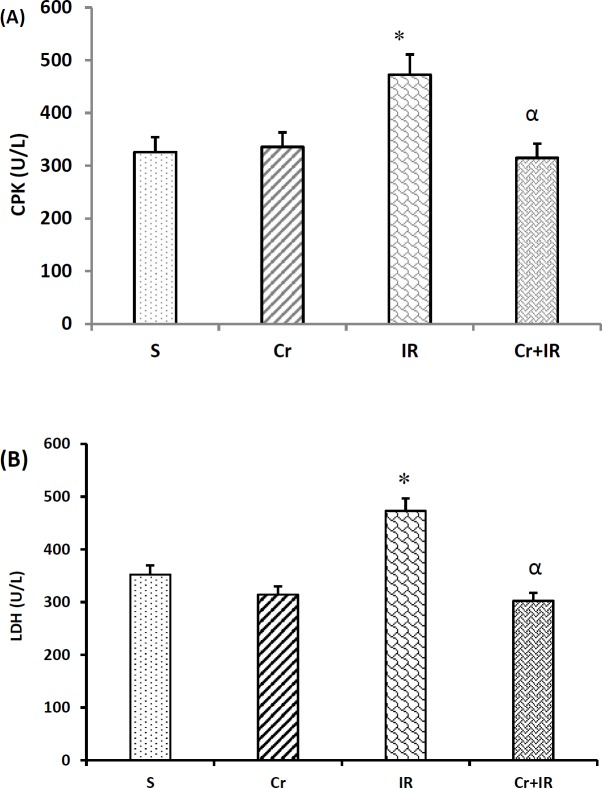
Crocin pretreatment at 200 mg/kg, on a daily basis for a week decreased CPK, and LDH concentrations in serum after IR-induced liver damage. Data represented as mean±SEM, 8 rats in each group. *P<0.05 shows significant increase comaped to the sham-operated rats and α*P*<0.05 demonstrated significant decrease compared to the IR rats. Cr: Crocin pretreatment; IR: Ischemia/reperfusion; S: Sham; Cr +IR: Crocin pretreatment+ ischemia/reperfusion; CPK: Creatine phosphokinase; LDH: Lactate dehydrogenase

**Figure 4:**
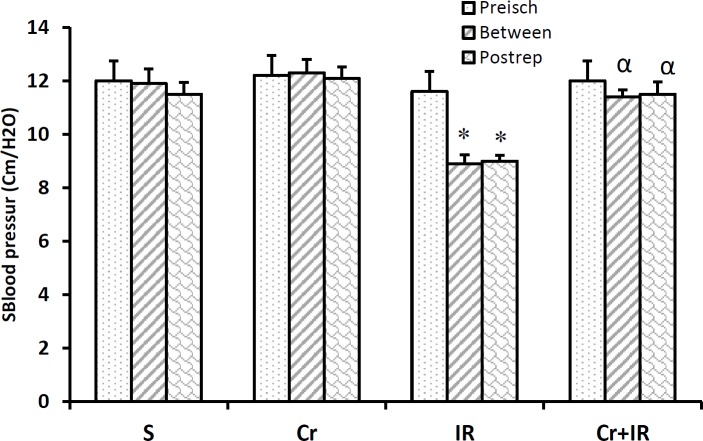
Crocin improved SBP after hepatic I/R injury. Results are represented as mean±SEM. *P<0.05 significant difference versus the sham-operated group and α*P*<0.05 significant difference versus the IR group. SBP: Systolic blood pressure; I/R: Ischemia/reperfusion; Preisch: Preischemia; Postrep: Postreperfusion

**Figure 5:**
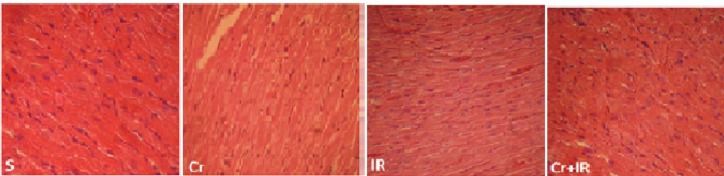
A representation of microscopic evaluation of heart tissue (magnification×300) of Hematoxylin and Eosin stained heart sections after liver IR damage. S: shows normal structure in sham rats; IR: shows moderate hemorrhage and congestion of blood vessel of myocardium in rats experienced hepatic ischemia/reperfusion (I/R) injury (blue arrow). Cr: shows normal architectural structure. Cr+IR: shows normal structure in rats pretreated with crocin before IR induction

**Table 1:** Crocin positively influenced the antioxidant activity beyond liver ischemia/reperfusion (I/R) damage. Data were expressed as mean±SEM. α*P*<0.05 versus the sham-operated group and β*P*<0.05 significant difference versus the IR group

**Experimental groups **	**Super oxide dismutase (U/ml)**	** Catalase (U/ml) **	**Glutathione peroxidase (U/ml)**
**Sham**	45.9	27.4	195
**IR**	^α^26.8	^α^14.5	^α^101
**Cr**	47.2	29.5	218.3
**Cr+IR**	^β^39	^β^26.8	^β^135.3

Cr: Crocin pretreatment; IR: Ischemia/reperfusion; Cr +IR: Crocin pretreatment+ ischemia/reperfusion

***Surgical procedure***

A mixture of ketamine [80 mg/kg, IP] (ketamine 10%, alfasan, woerden-holland) and xylazine [10 mg/kg, IP] (xylazin 2%, alfasan, woerden-holland) (14) were used to anesthetize the rats before surgery. After induction of anesthesia, a midline abdominal incision was generated and then liver ischemia was induced by occluding portal triad using a clamp artery. Duration of ischemia was 45 min, and then clamp was removed to establish reperfusion for 1 hr. Promptly changing the liver color to a pallor shade confirmed the correctness of ischemia (15). After finishing the time of reperfusion, samples of heart’s tissue and blood were taken for further histologic, enzymatic, and antioxidants evaluations.

***Determination of functional enzymes of liver and heart***

To measure the serum concentrations of hepatic transaminases and cardiac enzymes including creatine phosphokinase (CPK) and lactate dehydrogenase (LDH)], serum was separated by blood centrifugation (3000 rpm, 5 min). Afterwards, commercially kits (Pars Azmoon) were used to determine the serum levels of above parameters by using an autoanalyzer (BT-1500-A-A, Rome, Italy).

***Determination of the activity of antioxidants***

The activities of superoxide dismutase (SOD), catalase (CAT), and glutathione peroxidase (GP_X_) in heart’s homogenate were determined according to the following method. First, one milliliter of phosphate buffered saline (PBS, pH: 7.4) was added to 100 mg of each sample, then the mixture was homogenized at 14000 rpm for 3 min (16). After that, the homogenate was centrifuged at 12000 rpm at +4 ^°^C for 20 min. Following that, the supernatant phase was pipetted up and transferred in a new tube and used for measurement of the above mentioned parameters by specific kits (Zellbio GmbH, Germany) and finally read by micro plate/ELISA reader. 

***Histopathological analysis***

Formalin-fixed heart tissues were used to investigate the histological changes. Seventy two hour after fixation, samples were dehydrated using graded alcohol, embedded in paraffin, cut into 5 µm sections by a microtome (Leica RM 2125, Leica Microsystems Nussloch GmbH, Germany), and then stained with Hematoxylin and Eosin (8). Finally, a digital microscope was used to take images (BMZ-04-DZ, Behin Pajouhesh. ENG. CO. Iran). 

***Statistical analysis***

One-way analysis of variance (ANOVA) and Dennett’s tests were used to analyse the data. Data was represented as mean±standard errors of the mean (SEM). A significant difference of *P*<0.05 was considered statistically significant. 

## Results

***Effects of crocin on liver functional tests***

Figures 2A and 2B show that there were no differences between the serum concentrations of aspartate aminotransferase (AST), and alanine aminotransferase (ALT) in crocin-pretreated group with the sham-operated rats, while these levels significantly increased after induction of I/R injury in relative to the sham-operated group (*P*<0.05 and *P*<0.001, respectively). These figures also show that the serum levels of AST, and ALT in crocin-pretreated IR injury rats were less than those in IR group (*P*<0.05 and *P*<0.01, respectively). 

***Crocin effects on serum levels of CPK and LDH***

Figures 3A and 3B show that following partial hepatic IR injury, serum levels of CPK and LDH increased as compared to the sham-operated group. The concentration of CPK and LDH in serum in crocin- pretreated IR rats were less than those in IR group (*P*<0.01). 

***Crocin effects on the activity of antioxidant enzymes in heart homogenate***

Table 1 shows that hepatic IR injury decreased the activity of SOD, CAT, and GPx in heart homogenate in comparison with sham-operated rats (all cases *P*<0.05). This table also indicates that pretreatment with crocin effectively raised these levels compared to I/R group (*P*<0.05). 

***Crocin effects on blood pressure and heart rate after hepatic I/R injury***

As indicated in Figure 4, beyond partial liver IR injury, systolic blood pressure (SBP) decreased between the interval of ischemia, reperfusion, and postreperfusion times in relative to the sham-operated rats (*P*<0.05). Figure 4 showed that in crocin-pretreatment rats (200 mg/kg for 7 continual days, IP), SBP effectively increased in relative to IR rats at these studied time points (*P*<0.05). The impact of crocin on heart rate was not significant.

***Crocin pretreatment structurally preserved heart tissue after hepatic IR injury***

Figure 5 illustrated the microscopic changes such as mild hemorrhage and congestion in cardiac tissue following liver IR damage (blue arrow). Crocin pretreatment significantly but not completely prevented these alterations. As shown, heart tissue appeared normal in sham-operated and crocin groups. 

## Discussion

The current experiment showed that pretreatment with crocin inhibited IR-induced hepato-cardiac insult in rat via 1. Attenuating serum concentrations of AST and ALT; 2. Increasing the activity of antioxidant enzymes in cardiac tissue; 3. Preventing the architecture changes of cardiac; and 4. Modulating the hemodynamic parameters. Various studies have reported that cardiac dysfunction occur following major liver surgical protocols if the liver is subjected to a significant decrease in blood circulation or ischemia followed by reperfusion (2, 17).

It has been shown that several pathways are involved in pathophysiology of IR-induced cell insult. These pathways include disturbance in homeostasis of calcium in cells, absence of ATP, activation of phospholipases and proteases and production of active oxygen and nitrogen species that lead to oxidative stress (18).

According to evidences, the serum concentrations of liver transaminases increased after IR-induced hepatic damage (15, 19). A rise in these parameters represents hepatic cell damage, but these factors are not specific to this organ (20). The current study demonstrated a rise in the concentration of liver transaminases following liver IR injury in rats, and crocin inhibited these changes. The beneficial effects of crocin were also reported against nicotine-induced liver damage (8). Creatine kinase-MB (CK-MB) and LDH are released from cardiomyocytes into the extracellular fluid during myocardial damage. Therefore, they are considered as diagnostic enzymes. It has been reported that crocin pretreatment dramatically attenuated the levels of these enzymes in serum (21). Our study also indicated that hepatic IR injury led to an increase in the serum levels of these enzymes, which was significantly attenuated by crocin pretreatment. This effect of crocin may be achieved by its membrane-stabilizing effect (22), because of its potent antioxidant activity as proved by increasing the activity of antioxidant enzymes (23), which is confirmed by the limited extent of histological changes.

It has been reported that IR-induced hepatic insults accompany with cardiac cellular necrosis (24), which ROS production has significant role in this injury (25).This insult restored to near normal by administration of antioxidant (3). In this experiment, we indicated that the antioxidant activity is attenuated in the cardiac homogenate following IR-induced liver damage. In consistent with our findings, the results of one research showed that the levels of SOD and CAT gene expression were attenuated after oxidative stress (26). Another study also reported that crocin through increasing the activity of SOD and CAT protects the heart against IR injury (23). In a recent research, we found that the hepatoprotective effect of crocin on IR injury is partly mediated through increasing the activity of these antioxidant enzymes (9). In line with our results, these findings also showed the reproduction of antioxidant enzymes in heart homogenate after crocin administration.

As already demonstrated, IR leads to the release of apoptotic mediators, and ROS production, which may be involved in pathophysiology of cardiovascular hemodynamic disturbances (2). Furthermore, evidences have indicated that liver ischemia is associated with the release of adrenaline, noradrenaline, thromboxane A2 and angiotensin II. Besides, immediate inotropic action on the heart followed by a fast decrease in cardiac action was reported after reperfusion of the ischemic liver (2, 27). 

Our data represented that crocin pretreatment dramatically increased the reduced SBP beyond this intervention. Consistently, the modulatory actions of crocin on hemodynamic factors including left ventricular developed pressure (LVDP), coronary perfusion pressure, left ventricular systolic pressure, and myocardial contractility after IR damage was reported. These impacts may be attributed to its efficacy on potentiation of antioxidant capacity (28); in addition, crocin exerts antiarrhythmic effect on reperfusion-induced cardiac arrhythmias (29). 

## Conclusion

This evaluation indicated that crocin has potential cardioprotective action on IR-induced damage. Thus, it was suggested that this agent can be used before elective hepatic surgeries for inhibition of these side effects. 

## Conflicts of Interest

All authors declare that they have no conflicts of interest.
